# Comparison of malaria diagnostic methods in four hospitals in the Volta region of Ghana

**DOI:** 10.5281/zenodo.10797112

**Published:** 2016-06-10

**Authors:** Bismarck Dinko, Reuben Ayivor-Djanie, James Abugri, Eric Agboli, Gideon Kye-Duodu, Senyo Tagboto, John Tampuori, Festus Adzaku, Fred N. Binka, Gordon A. Awandare

**Affiliations:** 1 Department of Biomedical Sciences, School of Basic and Biomedical Sciences, University of Health and Allied Sciences, Ho, Ghana; 2 Department of Epidemiology and Biostatistics, School of Public Health, University of Health and Allied Sciences, Ho, Ghana; 3 Department of Biochemistry, Cell and Molecular Biology, University of Ghana, Legon, Accra, Ghana; 4 West African Centre for Cell Biology of Infectious Pathogens, University of Ghana, Legon, Accra, Ghana; 5 Department of Internal Medicine, School of Medicine, University of Health and Allied Sciences, Ho, Ghana; 6 Department of Urology, Volta Regional Hospital, Ho, Ghana

## Abstract

**Background:**

Rapid diagnostic tests (RDTs) and microscopy are routinely used for the diagnosis of malaria in Ghana. DNA-based polymerase chain reaction (PCR) is not yet used routinely. We compared diagnostic methods and tested the sensitivities of different malaria diagnostic methods against PCR.

**Materials and methods:**

Study participants from four hospitals with a suspicion of malaria donated finger -prick blood for RDT and blood film examination. In addition, a blood spot was collected for PCR analysis, prior to treatment. Retrospective species-specific PCR was performed on all samples collected.

**Results:**

Using PCR we found an overall malaria prevalence of 39% among the 211 evaluable blood spots (83/211) and this ranged between 6-61% across the four hospitals. Of the 164 participants with RDT data, malaria prevalence was 57% (94/164), ranging from 3-100% from the four hospitals. Microscopy was the least sensitive with a parasite prevalence of 21% (25/119) of the evaluable 119 participants, varying from 9 to 35% across three health facilities. By comparison, we found the sensitivities and specificities of RDT results when compared to PCR to be slightly higher than microscopy compared to PCR. These were 56.4% versus 41.7% and 90% versus 81.9%, respectively, but generally lower than expected. Ninety-five percent of the PCR-detected infections were *P. falciparum*, while 4% were mixed species infections of *P. falciparum* and *P. malariae*, with the remaining being a mono-infection of *P. malariae*.

**Conclusions:**

While using PCR as a gold standard, we found RDT to be more reliable in diagnosing malaria than microscopy. In addition, a majority of malaria-treated cases were not supported by PCR diagnosis, leading to possible overtreatment. Pragmatic strategies are needed to ensure suspected malaria cases are accurately diagnosed before treatment.

## 1 Introduction

Although reductions in malaria burden have been achieved across sub-Saharan Africa [1,2], the disease continues to be a challenge in various parts of Ghana, including the Volta region. In Ghana, malaria is responsible for 40% of outpatient attendances in public hospitals and 60% of these are children <5 years of age and pregnant women [3]. Microscopy is the ‘standard’ point-of-care malaria diagnostic method in Ghana. However, rapid diagnostic tests (RDTs) have recently been accepted as a good replacement or an addition to blood film examinations [4,5]. This has been particularly so in rural settings where there are insufficient trained microscopists [6,7]. Current RDTs detect either the *P. falciparum* histidine-rich protein 2 (Pf HRP2) or the L-lactate dehydrogenase (PfLDH) [4]. Prior to the availability of RDTs most clinicians relied on the signs and symptoms of malaria for diagnosis of suspected clinical infections [7-9]. In urban and peri-urban health facilities in Ghana, microscopy and RDT are now recommended for the diagnosis of suspected malaria cases [4, 5].

However, like microscopy, RDTs have limitations in identifying malaria infections [10-12], and in such cases only sensitive molecular techniques such as the polymerase chain reaction (PCR) can be used to determine the accuracies of these diagnoses. Furthermore, identification of malaria species causing clinical malaria during routine healthcare can be more accurately determined by PCR as compared to microscopy. A high number of non-falciparum malaria infections have been detected in asymptomatic school children in the Ashanti region of Ghana [13], and it is thought that non-falciparum malaria could have a major contribution to clinical malaria in certain parts of Ghana.

Despite the availability of these definitive diagnostic tools, presumptive treatment of malaria on the basis of clinical symptoms, with or without results of RDT and microscopy, is still being observed in Ghanaian hospitals [14], which results in overtreatment. In doing so, antimalarials are given to people who do not have malaria, and the malaria-negative individuals are being given inappropriate treatment for unidentified ailments [9,15]. Indiscriminate administration of anti-malarials can also contribute to the development of drug resistance [15].

This study investigated the reliability of microscopy and RDTs as malaria diagnostic methods for patients in four hospitals in the Volta region of Ghana, using PCR as a gold standard. PCR identification of parasite species was also performed to determine the contribution of non-falciparum species to clinical malaria in the region. These patients were clinically suspected of malaria, received prescription before laboratory diagnosis for confirmation as a routine in these health facilities.

## 2 Materials and methods

### 2.1 Study area and health facilities

Ethical approval to conduct this study was obtained from the Ghana Health Service ethics committee (GHS-ERC14/03/14), Accra. In addition, informed consent and/ or assent was obtained from study participants or their parents/guardians before enrolment in the study.

We performed a cross-sectional study on patients with a suspicion of malaria, in four hospitals in the Volta region. This region has the highest points in the country, and remains underserved with health facilities. The vegetation across this region is varied but consists mainly of tropical forest and forest savannah. The swamps within the forest areas support rice cultivation and other farming activities, which forms a major occupation of the people in this region. These rice fields form breeding grounds for malaria vectors [16]. There are two main rainy seasons, the major wet season lasting from April to July, and the minor wet season from September to November. Malaria is responsible for about 30% of hospital attendances in this region, 10% of which result in deaths each year [16].

The four study sites were Ho polyclinic, Ho municipal hospital, Volta regional hospital and Hohoe municipal hospital. The first three are the main public facilities within the Ho municipality. The Ho polyclinic provides mainly primary healthcare, whereas the Ho municipal hospital provides both primary and secondary healthcare. There is an average of four malaria cases per day in the Ho polyclinic, when considered cumulatively throughout the year [17]. The Volta regional hospital serves as a point for tertiary healthcare for the entire Volta region but routinely provides primary and secondary healthcare to patients. The Hohoe municipal hospital is the main healthcare provider and is located in Hohoe, which is the administrative capital of the municipality. In 2008, the malaria parasite rate was found to be 35%, and the entomological inoculation rate (EIR) was 165 infectious bites per person per year [16]. The major wet season in this area lags behind the peak of malaria transmission by about one month [16].

### 2.2 Study participants and sample collection

Individuals of varying ages that were clinically suspected to have malaria with a request for a malaria test and given consent were enrolled in the study. The criteria for the suspicion of clinical malaria included fever, headache, weakness/body ache and vomiting/nausea. Commonly, clinically suspected and diagnosed malaria cases presented at the laboratory with a prescription already written before the RDT and/or microscopy tests were carried out. The study was conducted during the months of February to August 2014. Following the routine healthcare systems in these hospitals, finger-prick blood samples were taken for blood film, RDT or both. An additional blood spot was taken on filter paper for PCR analysis. RDTs and blood smears were performed by laboratory staff within the respective health facilities. Filter-paper blood spots were dried in an air-conditioned room for 24 h and stored for subsequent molecular analyses.

### 2.3 RDTs and examination of blood smears

In all four hospitals, RDTs (Premier Medical Corporation, Gujarat, India) detecting histidine-rich protein 2 supplied by the Ghana Health Service were used to diagnose malaria. At the Ho polyclinic, RDT was the only method of diagnosis. However, in the Hohoe municipal hospital microscopy was used when RDTs were not available. Both microscopy and RDTs were used to diagnose malaria in the Ho municipal hospital and the Volta regional hospital. Thick and thin blood smears were stained with 10% Giem-sa and examined by microscope. Counts of parasitaemia were not made, patients were only assigned as malaria positive or negative.

### 2.4 Species detection by nested PCR

Filter-paper blood spots were carried to the Infectious Disease Research Laboratory within the Department of Biochemistry, Cell and Molecular Biology, University of Ghana, for DNA extraction and PCR analyses. In all our analyses, PCR was used as a gold standard for the detection of malaria parasites. Parasite DNA was extracted from filter paper blood spots in 96-well plates using Chelex resin as previously described [13,18]. *Plasmodium* species were detected and identified by nested PCR amplification of the small sub-unit ribosomal genes as previously described [19] with the second round PCR primers and amplicon sizes as in Dinko *et al*. [13]. For the second round amplification, 1 μl of first-round PCR product was amplified in a 20-μl reaction containing 10 μl of HotStar Taq master mix (Qiagen, UK) and 0.2 μM of each primer. PCR products were resolved in 2% agarose gels and visualised under UV light.

### 2.5 Calculation of sensitivity and specificity of RDT and microscopy compared to PCR

Sensitivity, specificity as well as the positive predictive values (PPV) and negative predictive values (NPV) were calculated using PCR as the gold standard. Sensitivity was equal to TP/(TP + FN) × 100%, and specificity was equal to TN/(TN + FP) × 100%. We calculated PPV using the formula TP/(TP + FP) × 100% , whereas the NPV was obtained from the formula TN/(TN + FN) × 100%. When comparing RDT and PCR, TP was the number of true positives (RDT and PCR positives), TN the number of true negatives (RDT and PCR negatives), FP the number of false positives (RDT negative and PCR positive) and FN the number of false negatives (RDT positive and PCR negative). Similar considerations were made when comparing microscopy with PCR.

### 2.6 Data analysis

Estimation of summary statistics was performed in STATA software (Stata 14.0, Statacorp, Texas, US). Microscopy and RDT were each compared with PCR by calculating Kappa values with 95% confidence intervals to assess the agreements between tests. These were done with Stata version 14 software.

## 3 Results

### 3.1 Parasite diagnosis by microscopy, RDT and PCR

On the basis of suspected clinical malaria, prescription of antimalarials and requests for malaria diagnosis, 211 (Ho polyclinic =71, Hohoe municipal hospital =69, Ho municipal hospital =36 and Volta regional hospital =35) individuals donated finger-prick blood samples for various kinds of malaria tests (Table 1). Forty-eight percent of the study participants in all four hospitals were children <5 years old, but the mean age of all participants was 11.6 years (range 1-81 years), with a male:female ratio of 98:110 (Table 1). The proportion of patients with prior antimalarial treatment two weeks before attending hospital was 18%.

**Table 1.** Char acteristics of enrolled patients at the four hospitals: Ho polyclinic, Hohoe municipal hospital, Ho municipal hospital and the Volta regional hospital.

	Ho polyclinic	Hohoe municipal hospital	Ho municipal hospital	Volta regional hospital	All 4 hospitals
N	71	69	36	35	211
Gender (M/F)	33/38	30/36	17/19	18/17	98/110*
Mean age/yr (range)	12.7 (1-61)	6.(1-42)	18.3 (1-81)	12.1 (1-45)	11.6 (1-81)
Reported fever (n/%)	51 (81%)	66 (100%)	33 (92%)	33 (94%)	183 (91.5%)

* Three participants had their gender details missing.

**Figure 1.**
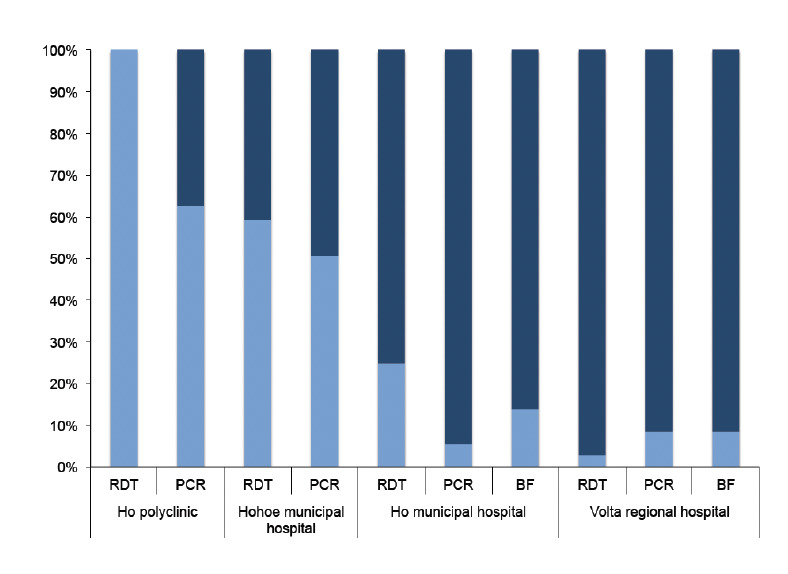
A comparison of the proportions of positive test results versus negative test results between RDT, PCR and BF diagnosis. All three methods were used in the Ho municipal hospital as well as the Volta regional hospital. In the Hohoe municipal hospital, RDTs were the main diagnostic method used, similar to the Ho polyclinic; microscopy was only performed in cases where RDTs were not available. In some cases, there were no agreements between two or all three diagnostic methods. RDT: rapid diagnostic test, PCR: polymerase chain reactions, BF: blood film for microscopy.

Twenty-one percent (25/119) of patients were positive for malaria by blood film (BF) in all four hospitals. The BF positivity rates for the respective hospitals were 35% (17/48) at the Hohoe municipal hospital, 14% (5/36) at the Ho municipal hospital and 9% (3/35) (Figure 1) at the Volta regional hospital. Of the 164 participants who had RDT data, 57% (94/164) of these tested positive for antigenae-mia detecting HRP2. Whereas all individuals tested with RDT were found to be positive for malaria at the Ho polyclinic, 59% (13/22) of the tests were positive at the Hohoe municipal hospital. We found 25% (9/36) of the individuals showing positive RDT results at the Ho municipal hospital while only 3% (1/35) (Figure 1) were found to be positive at the Volta regional hospital. Therefore, both through microscopy and RDT a substantial number of individuals presenting with clinical symptoms suggestive of malaria were found to test negative but were still given treatment against malaria. In addition, the positivity rate was higher when patients were diagnosed with RDT as compared to BF diagnosis.

Given the presumptive diagnosis and prescription of anti-malarials we aimed to use PCR as a gold standard to determine which of the enrolled individuals actually carried malaria parasites before treatment, regardless of the RDT and microscopy results. By PCR, we found an overall prevalence of 39% (83/211) of the 211 evaluable filter paper blood spots. The highest malaria prevalence by PCR was found at the Ho polyclinic to be 61% (43/71) with the Ho municipal hospital recording the lowest of 6% (2/36). At the Hohoe municipal and Volta regional hospitals PCR prevalence was found to be 51% (35/69) and 9% (3/35), respectively (Figure 1). Using PCR diagnosis, the level of misdiagnosis of malaria was highest at the Ho municipal hospital (94%) and lowest at the Ho polyclinic (39%).

### 3.2 Comparison of microscopy and RDT diagnostic methods to PCR

Out of the 118 samples with microscopy and PCR data, 10 samples were diagnosed positive by both methods while 77 of these participants were both microscopy and PCR negative. There were 14 samples determined by microscopy to be positive which in fact were PCR negative but 17 samples which were microscopy negative were found to positive by PCR as expected. Of the 164 samples with both RDT and PCR results, 53 tested positive for both tests. In addition, 63 individuals were negative by both tests, while 41 PCR negatives were found to be also RDT positive and 7 PCR positives were negative by RDT (Table 3). Therefore, there were often disagreements when comparing microscopy and RDT results to PCR. Further, Kappa values with 95% confidence intervals were determined to assess agreements between RDT tests with PCR on one hand and microscopy with PCR on the other hand. Generally, for all four hospitals, there was an agreement of 70.73% between RDT and PCR (Kappa value=0.43, p=0.0000) and 73.73% between microscopy and PCR (Kappa value=0.23, p=0.0071).

**Table 2.** Different age categories of study participants and the positivity rates of RDT versus PCR diagnosis across all four hospitals.

Age/yrs	N (%)	Positivity of RDT (%) rate	Positivity of PCR (%) rate
<5	102 (48%)	59	38
5-9	43 (20%)	65	49
10-16	14 (7%)	58	43
>16	52 (25%)	49	33
Total	211 (100%)		

**Table 3.** Performance of microscopy and RDT as diagnostic tests compared to PCR.

	Microscopy +	Microscopy -	Total	RDT +	RDT -	Total
PCR +	10	17	27	53	7	60
PCR -	14	77	91	41	63	104
Total	24	94	118	94	70	164

A pairwise comparison of the number of positives and negatives from microscopy and RDT results against PCR. There were often disagreements between the test methods as not all microscopy positives were RDT- or PCR+.

However, the data show slightly higher sensitivities and specificities of RDT when compared to PCR over microscopy compared to PCR, as 56.4% versus 41.7% and 90% versus 81.9%, respectively (Table 4). In addition, the possibility of diagnosing a sample to be positive also followed a similar pattern. Overall, the sensitivities and specificities of the diagnostic methods observed were much lower than the expected [20].

**Table 4.** Sensitivity and specificity of microscopy and RDT compared to PCR in all four hospitals.

	Sensitivity	Specificity	PPV*	NPV*
Test	(%)	(%)	(%)	(%)
Microscopy/PCR	41.7	81.9	37.0	84.6
RDT/PCR	56.4	90.0	88.3	60.6

*PPV: Positive Predictive Value. NPV: Negative Predictive Value.

### 3.3 Species identification by species specific PCR

Species-specific PCR based on ribosomal RNA (rRNA) small subunit (SSU) detected 39% of all PCR tested samples to be *P. falciparum* infections. Two percent (5/211) of all PCR tested cases were attributed to *P. malariae* and mixed infections between *P. falciparum* and *P. malariae*. These included one mono-infection of *P. malariae* from a patient attending the Hohoe municipal hospital. The remaining four infections were mixed species infections of *P. falciparum* and *P. malariae,* all from patients attending the Ho polyclinic. No *P. ovale* was detected in any of the hospitals and *P. vivax* was not included in the PCR analyses. We interrogated these five samples to find out if they were also positive by other tests methods and all except *P. malariae* mono-infection were positive by RDT from the respective hospital.

## 4 Discussion

In a region of stable malaria transmission in the Volta region of Ghana, 60% of malaria-treated cases in four hospitals were confirmed by PCR not to be malaria infections. RDTs had high sensitivity in detecting *P. falciparum* infections compared to PCR and microscopy. A majority of PCR-confirmed malaria cases were caused by *P. falcipa-rum* with *P. malariae* occurring as mixed infections with *P. falciparum.* In addition, a majority of test results between the different diagnostic methods were often discordant.

The high positivity of RDT over PCR (Table 2) in detecting malaria infections can be attributed to false RDT positives due to sub-standard RDT products, poor storage or misinterpretations of RDT results. Considering the fact that microscopy and RDT data were obtained from the various health facilities where there are highly trained laboratory personnel, it is unlikely that misinterpretation of the results was responsible for the high false positivity of RDTs observed. Therefore, the quality of RDTs available in the health system in Ghana as well as the storage conditions of these diagnostic tools needs to be evaluated.

Faye *et al*. [20], in a recent study carried out in Senegal, argued that the persistence of HRP-2 antigen in blood could account for high rates of RDT false positives. HRP-2 antigen-based RDTs often remain positive for more than five weeks after clearance of live malaria parasites due to the persistence of the HRP-2 antigen in remains of dead parasites [21]. This could be responsible for high false positive rates and low specificity of RDTs compared to microscopy or PCR, especially in high transmission settings [22,23]. Further, evaluations of RDTs based on HRP-2 have reported high sensitivities in medium to high malaria transmission settings where parasite densities often exceed 200 parasites/μl [10,24].

Although the absolute prevalence of malaria by all three diagnostic methods was slightly above half the number of enrolled individuals, all patients were treated with anti-malarials according to the national guidelines for the management of outpatients. Therefore, by estimation there was overtreatment of malaria by ~60%, taking into consideration PCR as gold standard. This was attributable to use of clinical symptoms alone as evidence of malaria infection.

Malaria overtreatment may lead to a number of consequences including failure to treat other serious causes of malaria related symptoms, such as fever [15,25,26], emergence of drug resistance, unnecessary adverse effects, reduced quality of healthcare and increased treatment cost [27,28]. Some studies have shown that mortality among children treated for malaria is over twofold higher in malaria-negative children than in children with laboratory-confirmed malaria, mostly as a result of ignored bacterial infections [15,25,29,30].

On the other hand, clinicians in countries with high malaria burdens may over-treat malaria because they do not trust negative test results or feared missing malaria cases [31], which could potentially be fatal. Thus, understanding malaria overtreatment is important in overcoming these serious potential consequences, and such information would improve upon the general management of all clinical infections and the understanding of the need to adhere to guidelines especially in resource poor settings [30].

We observed higher sensitivity and specificity when RDT was compared with PCR results when contrasted with microscopy versus PCR results. This is explained by the low sensitivity and specificity of blood film examination compared to RDT. This observation thus suggests that diagnosis based on RDTs is more reliable than microscopy. At the same time, diagnosis by RDT can sometimes lead to misdiagnosis and overtreatment, as a number of RDT-positive samples were PCR negative. It is worth noting that the current sensitivity and specificity are derived from PCR results and are therefore relative. In addition, one limitation of our study is that quantitative assessments of parasitaemia by PCR and microscopy were not performed due to resource constraints. These measurements will be carried out in a future study to bring the levels of parasitaemia into context with our comparative analyses.

In addition, the concordance between RDT and PCR (32%) was higher than the agreement between RDT and microscopy results (8%). This difference was expected since RDT and PCR are more sensitive in detecting malaria parasites than microscopy. A future study evaluating the accuracy of microscopy and RDT results in clinical malaria patients together with PCR where blood spots for PCR are obtained from RDT cassettes would enable us understand the differences in sensitivity and agreements between these diagnostic tools. In such a study it will be appropriate for the research team to carry out the microscopy and RDT investigations in addition to obtaining the corresponding test results from the health facilities.

Non-falciparum *Plasmodium* species have been previously described to contribute substantially to asymptomatic malaria infections in the Ahafo Ano south district of the Ashanti region of Ghana [13]. We found a small proportion of infections in the Volta region being accounted for by mixed species infections of *P. falciparum* and *P. malar-iae* and only a single mono-infection of non-falciparum species, *P. malariae*, was observed.

In this study, the diagnostic accuracy of RDT and microscopy under routine conditions were assessed against PCR as the gold standard. We chose PCR because it has the ability to detect low levels of parasitaemia in malaria patients, and with 100% sensitivity and specificity in patients with 5 or fewer parasites per microliter [19]. However, the exigency, and importance of obtaining test results for suspected symptomatic malaria patients limits the usefulness of conventional PCR for malaria diagnosis on the field. Furthermore, PCR is an expensive tool for routine diagnosis in the malaria-endemic areas with high transmission. Therefore, there is the need for evaluation of existing malaria diagnostic tools, on which development of strategies for improved diagnosis should be based.

## 5 Conclusions

Current efforts and strategies in the management of outpatient clinical malaria using case detection based on RDTs and microscopy in addition to clinical symptoms are important. The data show RDTs to be more reliable in diagnosing malaria than microscopy when both methods were compared to PCR. At the same time, our study raises questions regarding the quality of RDT test results in health facilities. Further studies are required to test the accuracy and compliance of definitive diagnostic test for detection and management of malaria infections in clinical settings.

## References

[1] O’Meara WP, Mangeni JN, Steketee R, Greenwood B (2010). Changes in the burden of malaria in sub-Saharan Africa.. Lancet Infect. Dis..

[2] Bhatt S, Weiss DJ, Cameron E, Bisanzio D (2015). The effect of malaria control on Plasmodium falciparum in Africa between 2000 and 2015.. Nature.

[3] Ghana national malaria report, NMCP, (2013).

[4] Ansah EK, Narh-Bana S, Epokor M, Akanpigbiam S (2010). Rapid testing for malaria in settings where microscopy is available and peripheral clinics where only presumptive treatment is available: a randomised controlled trial in Ghana.. BMJ.

[5] Baiden F, Malm K, Bart-Plange C, Hodgson A (2014). Shifting from presumptive to test-based management of malaria - technical basis and implications for malaria control in Ghana.. Ghana Med. J..

[6] Moerman F, Lengeler C, Chimumbwa J, Talisuna A (2003). The contribution of the health-care service to a sound and sustainable malaria-control policy.. Lancet Infect. Dis..

[7] Batwala V, Magnussen P, Nuwaha F (2010). Are rapid diagnostic tests more accurate in diagnosis of plasmodium falciparum malaria compared to microscopy at rural health centres?. Malar. J..

[8] Zurovac D, Midia B, Ochola SA, English M (2006). Microscopy and outpatient malaria case management among older children and adults in Kenya.. Trop. Med. Int. Health.

[9] Reyburn H, Mbakilwa H, Mwangi R, Mwerinde O (2007). Rapid diagnostic tests compared with malaria microscopy for guiding outpatient treatment of febrile illness in Tanzania: randomized trial.. BMJ.

[10] Hopkins H, Bebell L, Kambale W, Dokomajilar C (2008). Rapid diagnostic tests for malaria at sites of varying transmission intensity in Uganda.. J. Infect. Dis..

[11] Bell DR, Wilson DW, Martin LB (2005). False-positive results of a Plasmodium falciparum histidine-rich protein 2-detecting malaria rapid diagnostic test due to high sensitivity in a community with fluctuating low parasite density.. Am. J. Trop. Med. Hyg..

[12] Mens P, Spieker N, Omar S, Heijnen M (2007). Is molecular biology the best alternative for diagnosis of malaria to microscopy? A comparison between microscopy, antigen detection and molecular tests in rural Kenya and urban Tanzania.. Trop. Med. Int. Health.

[13] Dinko B, Oguike MC, Larbi JA, Bousema T (2013). Persistent detection of Plasmodium falciparum, P. malariae, P. ovale curtisi and P. ovale wallikeri after ACT treatment of asymptomatic Ghanaian school-children.. Int. J. Parasitol. Drugs Drug Resist..

[14] Ameme DK, Afari EA, Nyarko KM, Malm KL (2014). Direct observation of outpatient management of malaria in a rural Ghanaian district.. Pan Afr. Med J..

[15] Reyburn H, Mbatia R, Drakeley C, Carneiro I (2004). Over-diagnosis of malaria in patients with severe febrile illness in Tanzania: a prospective study.. BMJ.

[16] Kweku M, Liu D, Adjuik M, Binka F (2008). Seasonal intermittent preventive treatment for the prevention of anaemia and malaria in Ghanaian children: a randomized, placebo controlled trial.. PLoS One.

[17] Volta regional malaria report, (2014).

[18] Oguike MC, Betson M., Burke M, Nolder D (2011). Plasmodium ovale curtisi and Plasmodium ovale wallikeri circulate simultaneously in African communities.. Int. J. Parasitol..

[19] Snounou G, Viriyakosol S, Jarra W, Thaithong S (1993). Identification of the four human malaria parasite species in field samples by the polymerase chain reaction and detection of a high prevalence of mixed infections.. Mol. Bio-chem. Parasitol..

[20] Faye B, Nath-Chowdhury M, Tine RC, Ndiaye JL (2013). Accuracy of HRP2 RDT (Malaria Antigen P.f®) compared to microscopy and PCR for malaria diagnosis in Senegal..

[21] Swarthout TD, Counihan H, Senga RK, van den Broek I (2007). Paracheck-Pf accuracy and recently treated Plasmodium falciparum infections: is there a risk of over-diagnosis?. Malar. J..

[22] Abeku TA, Kristan M, Jones C, Beard J (2008). Determinants of the accuracy of rapid diagnostic tests in malaria case management: evidence from low and moderate transmission settings in the East African highlands.. Malar. J..

[23] Kyabayinze DJ, Tibenderana JK, Odong GW, Rwakimari JB (2008). Operational accuracy and comparative persistent antigenicity of HRP2 rapid diagnostic tests for Plasmodium falciparum malaria in a hyperendemic region of Uganda.. Malar. J..

[24] Moonasar D, Goga AE, Kruger PS, La Cock C (2009). Field evaluation of a malaria rapid diagnostic test (ICT Pf).. S. Afr. Med. J..

[25] Gwer S, Newton CR, Berkley JA (2007). Over-diagnosis and co-morbidity of severe malaria in African children: a guide for clinicians.. Am. J. Trop. Med. Hyg..

[26] Stoler J, Delimini RK, Bonney JH, Oduro AR (2015). Evidence of recent dengue exposure among malaria parasite-positive children in three urban centers in Ghana.. Am. J. Trop. Med. Hyg..

[27] Amexo M, Tolhurst R, Barnish G, Bates I (2004). Malaria misdiagnosis: effects on the poor and vulnerable.. Lancet.

[28] Osei-Kwakye K, Asante KP, Mahama E, Apanga S (2013). The benefits or otherwise of managing malaria cases with or without laboratory diagnosis: the experience in a district hospital in Ghana.. PLoS One.

[29] Evans JA, Adusei A, Timmann C, May J (2004). High mortality of infant bacteraemia clinically indistinguishable from severe malaria.. QJM.

[30] Onchiri FM, Pavlinac PB, Singa BO, Naulikha JM (2015). Frequency and correlates of malaria over-treatment in areas of differing malaria transmission: a cross-sectional study in rural Western Kenya.. Malar. J..

[31] Odaga J, Sinclair D, Lokong JA, Donegan S (2014). Rapid diagnostic tests versus clinical diagnosis for managing people with fever in malaria endemic settings.. Cochrane Database Syst. Rev..

